# A Life-Course Study on Effects of Parental Markers of Morbidity and Mortality on Offspring’s Suicide Attempt

**DOI:** 10.1371/journal.pone.0051585

**Published:** 2012-12-12

**Authors:** Ellenor Mittendorfer-Rutz, Finn Rasmussen, Theis Lange

**Affiliations:** 1 Department of Clinical Neuroscience, Division of Insurance Medicine, Karolinska Institutet, Stockholm, Sweden; 2 Department of Public Health Sciences, Child and Adolescent Public Health Epidemiology, Karolinska Institutet, Stockholm, Sweden; 3 Department of Biostatistics, University of Copenhagen, Copenhagen, Denmark; Maastricht University Medical Centre, The Netherlands

## Abstract

**Background:**

Research on the temporal relationship of parental risk factors with offspring’s suicide attempt is scarce and a life course approach has not been applied to date. We investigated the temporal relationship of parental morbidity and mortality with offspring’s suicide attempt and whether any such association was modified by offspring’s age at attempt.

**Methods:**

We designed a case-control study through linkage of Swedish registers. Cases comprised all individuals in Sweden born 1973–1983 with inpatient care due to suicide attempt (15–31 years of age) and with information on both biological parents (N = 15 193). Ten controls were matched to each case (National Patient register with national complete coverage). Conditional logistic and spline regressions were applied.

**Results:**

Particularly for women, most parental markers showed the strongest effect sizes if exposure was short-term (within 2 years after exposure) and related to the mother. Especially short-term exposure to maternal inpatient care due to psychiatric diagnoses had a significantly stronger effect on suicide attempt risk in women compared to men. Regarding exposure to parental inpatient care due to psychiatric diagnoses, short-term as opposed to long-term (exceeding 2 years after exposure) effects were highest during adolescence and decreased significantly with age for female and male offspring, respectively.

**Conclusions:**

Although limited by the fact that data on parental morbidity and the outcome of suicidality were based on in-patient data only, the data suggest that the high risks of suicide attempt in case of exposure to parental psychopathology and suicidal behavior particularly during adolescence and the strong short-term effects associated with maternal psychopathology for female offspring are of direct clinical importance.

## Introduction

Suicide attempt is a considerable and growing public health problem worldwide [1], [2], [3]. Data from the WHO/EURO multicentre study revealed that young women (15 to 24 years) comprised the only age group with increasing suicide attempt rates in several centers studied between 1989 and 1999 [1], [4]. Also in countries with available data with national coverage on hospital admission due to attempted suicide, like Sweden, considerable increases of inpatient care due to suicide attempt among women between 15 and 24 years of age have been reported between the mid 1990s and 2007 [2], [5].

There is abundant evidence on the effect of familial suicidal behavior and familial psychopathology on the offspring’s risk of suicide and suicide attempt [6], [7], [8], [9], [10]. Recently two register studies in Sweden could demonstrate that the effect on the child regarding the risk for suicide gets more detrimental the earlier in life a maternal or paternal suicide occurred [11], [12]. Similar associations could be shown with regard to the timing of parental psychiatric inpatient care on offspring’s risk for suicide attempt [12]. According to the paradigm of life-course epidemiology, exposure during critical or sensitive periods in life, like for exemple childhood or adolescence, can have a more detrimental effect than if exposure occurs outside these time windows [13]. Particularly adolescence is known to be associated with a higher degree of impulsive behavior and emotional turmoil than adulthood [14]. For that reason we wanted to test if effects of exposure to parental morbidity and mortality vary with regard to timing of exposure and with regard to age at offspring’s suicide attempt.

These patterns of increased risk of suicidal behavior in offspring with decreasing age at exposure were shown not to be equally consistent regarding exposure to all types of parental markers of morbidity and mortality [12]. In case of the effect of parental death other than suicide, estimates for offspring’s suicidal behavior were highest if exposure occurred later in life (after the 10^th^ birthday as compared to before) [12]. These findings suggest short-term or triggering effects of parental risk factors on offspring’s suicidal behavior. In fact recent studies reported higher short-term than long-term effects of exposure to parental psychiatric morbidity for offspring’s suicide [15] and exposure to parental death for offspring’s suicide attempt [16]. To date, however, it is unclear as to whether these effects are similar for a wide range of parental exposures and if these short and long term effects vary with age at offspring’s suicide attempt.

Suicide attempt is reported to be more common among young women than among young men [1]. Different studies have suggested stronger effects of familial psychiatric morbidity and suicide on the risk of suicide for women as compared to men [7], [15], where else the risk for attempting suicide in case of familial suicide completion was found to be higher for boys than for girls [6]. Also, young women are reported to have higher rates of psychopathology than young men [5]. In addition, the effect of parental markers of morbidity and mortality are likely to be modified by offspring’s psychopathology [6]. Furthermore, higher risk estimates of maternal as compared to paternal suicide [17] and suicide attempt [6] for offspring’s risk of suicide attempt were reported. For these reasons, it is important to study the specific effects of maternal and paternal risk factors separately on female and male offspring when simultaneously considering the offspring’s own psychopathology.

The aims of the present study were therefore three-fold: (i) to explore short- and long-term effects of parental markers of morbidity and mortality on the risk of suicide attempt in offspring; (ii) to investigate whether exposure to these parental markers before age 10 confers an increased risk of suicide attempt compared to exposure above this age; and (iii) to investigate whether any such effect is modified by age of offspring at attempted suicide. To the best of our knowledge, this study is the first to analyse these research questions using a very large database including more than 15 000 suicide attempters.

## Methods

### Ethical Statement

The study population was based on linkage of several public national registers. Ethical vetting is always required when using register data in Sweden. The ethical vetting is performed by regional ethical review boards and the risk appraisal associated with the Law on Public Disclosure and Secrecy is done by data owners. For this study, the ethical review board has however waived the requirement to consult the data subjects (and in case of minors/children the next of kin, careers or guardians) directly to obtain their informed consent, as the research is supported by the ethical review board and the data has already been collected in some other context. According to these standards in Sweden this project has been evaluated and approved by the Regional Ethical Review Board of Karolinska Institutet, Stockholm, Sweden.

### Study Design

The study applied a matched case control study design through record linkages. The study base consisted of all individuals, born in Sweden between January 1973 and December 1983, who were singletons and for whom information on both biological parents was available. The cases comprised individuals with inpatient care due to attempted suicide recorded in the National Patient Register (NPR) as (E950–E959 in the International Classification of Diseases ICD-8 and ICD-9, X60–X84 in ICD-10).

Even cases with inpatient care due to uncertainty about intention (E980–E989 in ICD-8 and ICD-9, Y10–Y34 in ICD-10) were considered. Uncertain and certain diagnoses were combined to limit temporal and regional variation in ascertainment routines and limit the underreporting of suicidal behavior [18], [19]. A sensitivity analysis of certain and uncertain suicide attempt established the comparability of the estimates. This combined outcome measure is hereafter referred to as “suicide attempt”.

Up to 10 controls were randomly selected from a cohort covering all individuals born in Sweden between 1973 and 1983 and matched to cases by sex, month, year and county of birth. Only individuals who were alive and living in Sweden at the time of the index event were eligible to serve as controls. Suicide attempters were assessed from January 1, 1988 until December 31, 2006, and were between 15 and 31 years of age.

### Data Sources

Individual information has been merged from eight different national registers using the unique identification number assigned to each resident in Sweden. Children were linked to their biological parents using the Multi-Generation Register (MGR). The MGR is held by Statistics Sweden and contains links of children to their parents.

Data on suicide and other causes of death were drawn from the Causes of Death Register (CDR). Suicide was defined through the same codes in ICD as suicide attempt. Information on the dates and diagnoses of hospital care due to psychiatric diagnoses were derived from the National Patient Register (NPR). The NPR has complete national coverage on inpatient care due to psychiatric diagnoses and suicide attempt derived from general and psychiatric hospitals since 1987, and close to complete national coverage since 1973 [20]. The Register of the Social Insurance Agency provided information on diagnosis-specific disability pension. All diagnoses in the respective registers were classified in accordance with ICD-8, ICD-9 and ICD-10.

The Population and Housing Censuses (PHC) provided data on parental socio-economic status in 1980 and 1985 and on parental civil status in 1975, 1980 and 1985. Data on these covariates in 1990 were retrieved from the Longitudinal Integration Database for Health Insurance and Labor Market Studies (LISA). Information on emigration and immigration was extracted from the Register of the Total Population. Details of the registers as well as quality evaluations have been reported elsewhere [12].

### Exposure Variables

The exposure variables include parental markers of morbidity, namely inpatient care dut to suicide attempt and/or due to psychiatric disorders and diagnosis-specific disability pension as well as mortality (suicide and other causes of death). We considered only the main diagnosis, and the first date of hospital inpatient care or granting of disability pension. Data on all exposure variables were available from birth of the offspring until the end of follow-up.

### Covariates

Categories for parental socio-economic status were unskilled workers, skilled workers, low level salaried employees, intermediate or high level salaried employees (reference category), and others (Table 1). We used either maternal or paternal socio-economic status, whichever was the higher. This is in line with the dominance principle, which was reported to perform well in classifying the social status of families [21]. Data from the 1980 census were used for events of suicide attempt that occurred between 1983 and 1990, 1985 census data again for events between 1991 and 1998, and 1990 data for events from 1999 up until 2005. In the case of internal missing data, we took information from the preceding census in line with previous studies [12], thus ensuring that all measurements were taken at a point in time preceding offspring’s suicide attempt.

**Table 1:** Descriptive statistics of cases (9 748 girls/women and 5 445 boys/men) and of controls (92 862 girls/women and 53 362 boys/men).

	Girls, women, N, %				Boys/men, N, %			
Parental factors	Cases		Controls		Cases		Controls	
*Civil status*								
Married/cohabiting	5 588	(57.32)	67 465	(72.65)	3 011	(55.30)	38 849	(72.80)
Other	4 117	(42.23)	25 216	(27.15)	2 397	(44.02)	14 381	(26.95)
Missing	43	(0.44)	181	(0.19)	37	(0.68)	132	(0.25)
*Socio-economic status*								
Unskilled workers	2 086	(21.40)	14 913	(16.06)	1 291	(23.71)	8 443	(15.82)
Skilled workers	2 130	(21.85)	18 714	(20.15)	1 187	(21.80)	10 769	(20.18)
Low salary**	1 479	(15.17)	15 753	(16.96)	793	(14.56)	8 752	(16.40)
Med./high salary**	3 155	(32.37)	39 035	(42.04)	1 641	(30.14)	22 993	(43.09)
Other	889	(9.12)	4 429	(4.77)	527	(9.68)	2 388	(4.48)
Missing	9	(0.09)	18	(0.02)	6	(0.11)	17	(0.03)
*Events ever*								
Suicide	223	(2.29)	861	(0.93)	138	(2.53)	513	(0.96)
**Maternal events**								
Suicide ettempt	736	(7.55)	2 043	(2.20)	418	(7.68)	1 196	(2.24)
Psych. diagnosis*	1 550	(15.90)	5 477	(5.90)	882	(16.20)	3 370	(6.32)
Death non-suicide	220	(2.26)	1 510	(1.63)	160	(2.94)	921	(1.73)
DP psychiatr.	582	(5.97)	2 204	(2.37)	395	(7.25)	1 489	(2.79)
DP somatic	1 126	(11.55)	6 993	(7.53)	738	(13.55)	4 807	(9.01)
**Paternal events**								
Suicide attempt	481	(4.93)	1 691	(1.82)	332	(6.10)	993	(1.86)
Psych. diagn.*	1 469	(15.07)	6 283	(6.77)	963	(17.69)	3 768	(7.06)
Death non-suicide	454	(4.66)	2 955	(3.18)	338	(6.21)	1 970	(3.69)
DP psychiatr.	439	(4.50)	1 786	(1.92)	308	(5.66)	1 166	(2.19)
DP somatic	855	(8.77)	5 338	(5.75)	552	(10.14)	3 575	(6.70)
**Offspring events**								
Psych. diagnosis*	5 197	(53.3)	2 966	(3.2)	2 943	(54.1)	1 859	(3.5)

DP…disability pension (psychiatric/somatic diagnoses);

* Inpatien care due to a psychiatric diagnosis;

** employees.

Parental civil status was dichotomized into married and/or cohabiting versus other status (reference category), using the same procedure as for socio-economic status with regard to the choice of census data. Information on offspring’s own inpatient care due to psychiatric diagnoses preceding the suicide attempt was dichotomized. Data on covariates were retrieved in the same way for both cases and controls. There were missing data on covariates in 0.5% of cases of attempted suicide. A sensitivity analysis showed similar patterns of suicide attempt risks in cases with complete information and in cases with missing data. Cases with missing data were included and coded as a separate category.

### Statistical Analyses

Data processing was performed using R version 2.12.2. The analyses were based on conditional logistic regressions. The risk of different parental exposures (short- and long-term and exposure during childhood) in relation to suicide attempt in offspring was estimated comparing cases and controls. Short term was defined as less than 2 years from exposure to occurrence, long term exceeding 2 years since exposure, and exposure during childhood was defined as exposure occurring before the 10^th^ birthday of the offspring. The cut-off of 2 years has been chosen in accordance with other studies in this field [15], [16] and so that adequate power for the analyses could be guaranteed. Both crude and multiple adjusted Odds ratios (ORs) with 95% confidence intervals (CI) were calculated for all short and long term and childhood exposures to maternal and paternal risk factors for female and male offspring. Due to limited statistical power we combined maternal and paternal suicides. Similarly, the exposure variables maternal and paternal death due to other reasons than suicide could not be estimated during childhood.

To accommodate that the effect of a given exposure might be modified by age of offspring at time of attempted suicide, we applied spline regression with four knots (on 17, 21, 25 and, 29 years of age) for both baseline risk and exposures. [22], [23] This approach allowed both baseline risk and effect of exposure to change with age and made it possible to determine the shape of modifying age effects. We used the likelihood ratio test to test gender differences and deviation from­ a constant effect of the exposures across age. Following the principals described by Baron and Kenny, mediation was assessed by further adjusting for individual inpatient care due to psychiatric diagnoses (a potential mediator) and observed how the estimated effects change. Decreasing odds ratios (ORs) for a given exposure indicate that some of the effect of this exposure is mediated through individual inpatient care due to psychiatric diagnoses [24]. We also calculated a modified proportion when considering the effect of offspring’s inpatient care due to psychiatric diagnoses: Mediated proportion [LN (OR without mediator) - LN (OR with mediator) ]/LN (OR (without mediator) [25]. Recent work on causal mediation analysis [26] has shown that the Baron & Kenny method is only an approximation when used with logistic regressions. However, as the outcome (suicide attempt) is quite rare, there would be very little gained by employing the more modern methods for assessing mediation. Plots of odds ratios (ORs) with 95% Confidence Intervals (CIs) are presented across age for those exposure variables found to be statistically significantly age dependent. These plots include a dotted line indicating the effect when controlling for offspring’s inpatient care due to psychiatric diagnoses.

**Figure 1:**
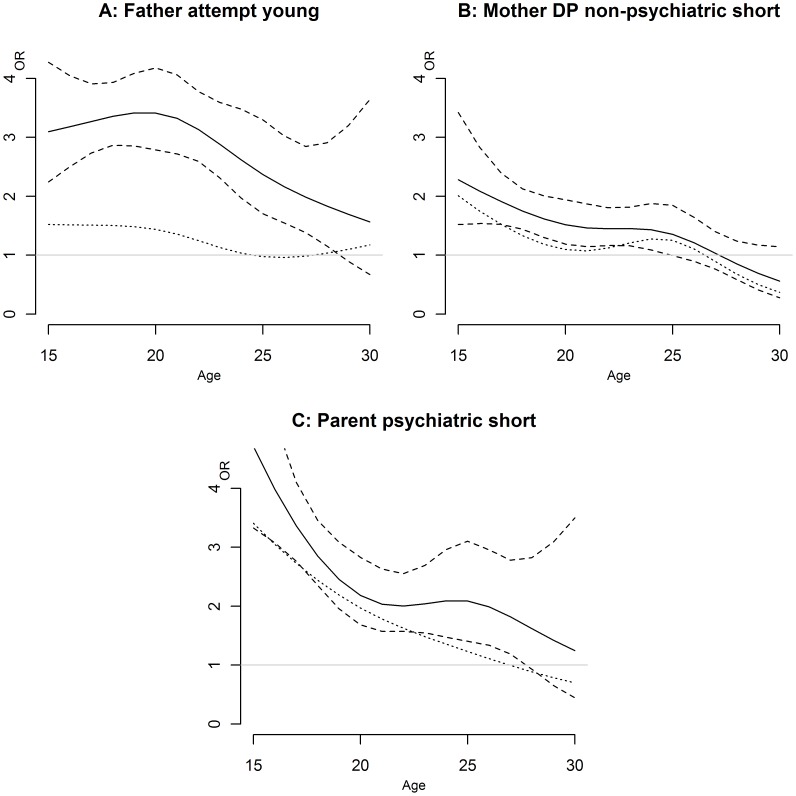
Effect modification by age of short term effects (less than 2 years) of exposure to parental morbidity on offspring’s suicide attempt, young women (natural cubic splines)*. The dotted line represents the ORs after controlling for offspring’s own inpatient care due to psychiatric diagnoses; *adjusted for parental socioeconomic status, civil status, suicidal behaviour, inpatient care due to psychiatric diagnoses, death due to other reasons than suicide and parental disability pension; DP…disability pension; “young”: refers to exposure to parental markers of morbidity and mortality occurring before the 10^th^ birthday of the offspring; “short”: Short-term was defined as an offspring’s suicide attempt occurring less than 2 years after exposure to parental markers of morbidity and mortality; “long”: Long-term was defined as an offspring’s suicide attempt occurring later than 2 years after exposure to parental markers of morbidity and mortality; “Attempt”: Inpatient care due to suicide attempt; “Parent psychiatric”: At least one parent in inpatient care due to a psychiatric diagnosis.

**Table 2:** Odds ratios and 95% confidence interval (95% CI) of suicide attempt in young women in relation to timing of exposure to parental markers of morbidity and mortality.

Exposure	N		Crude		Model I		Model II		Model III
Parental factors			OR (95% CI)						
Suicide early	102		2.57 (2.07–3.21)	1.59 (1.27–1.99)		1.37 (1.09–1.73)		0.99 (0.73–1.33)	
Suicide short	39		2.89 (2.02–4.15)	2.48 (1.72–3.57)		1.60 (1.11–2.33)		1.91 (1.21–3.00)	
Suicide long	184		2.45 (2.08–2.88)	1.70 (1.44–2.01)		1.38 (1.16–1.63)		1.03 (0.82–1.28)	
**Maternal factors**									
Attempt early		453	3.49 (3.13–3.89)	2.64 (2.36–2.95)		1.59 (1.41–1.80)		1.57 (1.35–1.83)*	
Attempt short		235	4.10 (2.92–5.76)	3.66 (2.59–5.17)		1.98 (1.39–2.82)*		1.98 (1.26–3.12)	
Attempt long		48	3.53 (3.04–4.11)	2.87 (2.46–3.34)		1.55 (1.32–1.82)		1.54 (1.25–1.89)	
Psych. early		981	3.02 (2.80–3.25)	2.41 (2.23–2.59)		1.72 (1.58–1.88)		1.29 (1.16–1.44)*	
Psych. short		132	3.38 (2.77–4.13)	3.17 (2.58–3.88)		2.58 (2.09–3.18)		2.38 (1.84–3.09)	
Psych. long		437	2.45 (2.21–2.73)	2.10 (1.89–2.34)		1.52 (1.36–1.70)		1.31 (1.14–1.52)	
Death short		66	1.64 (1.26–2.13)	1.49 (1.15–1.95)		1.28 (0.97–1.67)		1.16 (0.82–1.63)	
Death long		154	1.35 (1.14–1.59)	1.13 (0.94–1.35)		1.01 (0.84–1.21)		0.88 (0.70–1.11)	
**Paternal factors**									
Attempt early		335	3.03 (2.68–3.44)	2.18 (1.92–2.48)		1.46 (1.28–1.67)*		1.34 (1.13–1.59)	
Attempt short		111	3.17 (2.16–4.64)	2.65 (1.79–3.91)		1.63 (1.10–2.43)		1.67 (1.02–2.74)	
Attempt long		35	2.13 (1.73–2.62)	1.71 (1.38–2.10)		1.07 (0.89–1.36)		1.03 (0.78–1.36)	
Psych. early		992	2.75 (2.55–2.96)	2.05 (1.89–2.21)		1.67 (1.53–1.82)		1.41 (1.26–1.56)	
Psych. short		92	1.94 (1.55–2.43)*	1.68 (1.34–2.11)*		1.53 (1.22–1.92)*		1.49 (1.12–1.97)*	
Psych. long		385	1.78 (1.59–1.99)	1.51 (1.35–1.69)		1.27 (1.14–1.43)		1.22 (1.06–1.41)*	
Death short		111	1.62 (1.32–1.98)	1.44 (1.17–1.76)		1.20 (0.98–1.48)		1.29 (1.00–1.66)	
Death long		343	1.48 (1.31–1.66)	1.08 (0.96–1.22)		1.01 (0.89–1.14)		0.88 (0.76–1.02)	

Model I: adjusted for parental socioeconomic and civil status; Model II: like Model I and additionally adjusted for parental suicidal behaviour, parental inpatient care due to psychiatric diagnoses, disability pension and death due to other reasons than suicide; Model III: like Model II and additionally adjusted for offspring’s inpatient care due to psychiatric diagnoses prior to the index suicide attempt; *significant age dependent effects; “early”: refers to exposure to parental markers of morbidity and mortality occurring before the 10^th^ birthday of the offspring; “short”: Short term was defined as an offspring’s suicide attempt occurring less than 2 years after exposure to parental markers of morbidity and mortality; “long”: Long term was defined as an offspring’s suicide attempt occurring later than 2 years after exposure to parental markers of morbidity and mortality; “Psych.”: Inpatien care due to a psychiatric diagnosis; “Attempt”: Inpatient care due to suicide attempt; “Death”: Death due to reasones other than suicide.

**Table 3:** Odds ratios and 95% Confidence Interval (95% CI) of suicide attempt in young men in relation to timing of exposure to parental markers of morbidity and mortality.

Exposure		N			Crude		Model I	Model II		Model III	
Parental factors					OR (95% CI)						
Suicide early		60			3.01 (2.25–4.03)	1.64 (1.21–2.22)			1.41 (1.04–1.92)		1.06 (0.72–1.57)
Suicide short		15			2.29 (1.30–4.01)	1.82 (1.03–3.23)			1.21 (0.68–2.17)		1.05 (0.51–2.18)
Suicide long		124			2.76 (2.26–3.37)	1.79 (1.46–2.21)			1.44 (1.16–1.78)		1.21 (0.92–1.58)
**Maternal factors**											
Attempt early			237		3.45 (2.97–4.01)	2.44 (2.09–2.84)			1.55 (1.31–1.82)		1.34 (1.08–1.66)
Attempt short			149		4.13 (2.73–6.25)	3.46 (2.26–5.29)			2.07 (1.34–3.20)		2.33 (1.35–4.02)
Attempt long			48		3.54 (2.93–4.27)	2.78 (2.29–3.38)			1.70 (1.39–2.08)		1.57 (1.21–2.04)
Psych. early	555			2.97 (2.69–3.28)*		2.23 (2.02–2.47)*			1.62 (1.44–1.81)*		1.12 (0.97–1.29)
Psych. short			51		2.00 (1.48–2.71)	1.72 (1.27–2.34)			1.38 (1.01–1.88)		1.33 (0.91–1.94)
Psych. long			276		2.44 (2.13–2.79)	2.01 (1.76–2.31)			1.47 (1.28–1.70)*		1.25 (1.04–1.49)*
Death short			44		1.90 (1.38–2.64)	1.85 (1.33–2.57)			1.53 (1.09–2.13)		1.41 (0.93–2.15)
Death long			116		1.67 (1.37–2.04)	1.29 (1.04–1.61)			1.18 (0.95–1.47)		1.19 (0.91–1.55)
**Paternal factors**											
Attempt early			221		3.82 (3.26–4.47)	2.66 (2.26–3.12)			1.70 (1.44–2.03)		1.21 (0.97–1.52)*
Attempt short			92		3.79 (2.23–6.45)	2.92 (1.69–5.01)			1.73 (1.00–3.00)		1.35 (0.65–2.79)
Attempt long			19		2.56 (2.03–3.23)	1.92 (1.52–2.43)			1.15 (0.91–1.47)		0.83 (0.61–1.13)
Psych. early			630		3.17 (2.88–3.48)	2.25 (2.04–2.48)			1.76 (1.58–1.96)		1.54 (1.35–1.77)
Psych. short			50		1.93 (1.42–2.61)	1.72 (1.26–2.35)			1.58 (1.15–2.15)		1.29 (0.88–1.90)
Psych. long			283		2.07 (1.82–2.36)	1.69 (1.48–1.93)			1.41 (1.23–1.61)*		1.27 (1.07–1.51)*
Death short			71		1.59 (1.24–2.06)	1.38 (1.07–1.79)			1.13 (0.87–1.46)		1.14 (0.83–1.56)
Death long			267		1.77 (1.55–2.02)	1.25 (1.09–1.44)			1.16 (1.01–1.33)		1.12 (0.95–1.33)

Model I: adjusted for parental socioeconomic and civil status; Model II: like Model I and additionally adjusted for parental suicidal behaviour, parental inpatient care due to psychiatric diagnoses, disability pension and death due to other reasons than suicide; Model III: like Model II and additionally adjusted for offspring’s inpatient care due to psychiatric diagnoses prior to the index suicide attempt; *significant age dependent effects; “early”: refers to exposure to parental markers of morbidity and mortality occurring before the 10^th^ birthday of the offspring; “short”: Short term was defined as an offspring’s suicide attempt occurring less than 2 years after exposure to parental markers of morbidity and mortality; “long”: Long term was defined as an offspring’s suicide attempt occurring later than 2 years after exposure to parental markers of morbidity and mortality; “Psych.”: Inpatien care due to a psychiatric diagnosis; “Attempt”: Inpatient care due to suicide attempt; “Death”: Death due to reasones other than suicide.

## Results

### General Description

There were 9 748 attempted suicides (64.2%) in girls/women and 5 445 (35.8%) in boys/men in the given sample. Mean age at suicide attempt was 20.8 years (SD: 3.8) for girls/women and 22.2 years (SD: 3.8) for boys/men. The main method for suicide attempt with certain intent (N = 12,563) was poisoning (61.6%) followed by cutting (2.2%). Even in uncertain suicide attempts (N = 2,630), the main method was poisoning (77.1%). Table 1 presents an overview of the characteristics of cases and controls. A lower proportion of cases than controls grew up with parents being medium or high salary employees and being married or cohabiting. Exposure to maternal and paternal events (like suicide attempt and suicide, inpatient care due to psychiatric diagnoses and disability pension due to psychiatric diagnoses) was two to three times more frequent among both female and male cases than among controls. Previous inpatient care due to psychiatric diagnoses was much more common in cases than controls.

**Figure 2:**
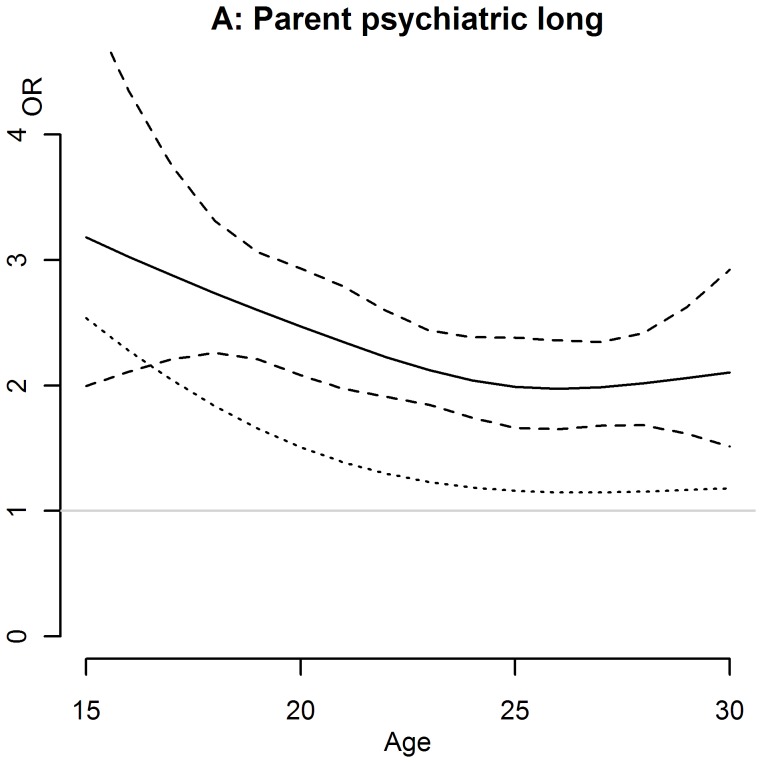
Trends of long term effects of exposure to parental psychiatric morbidity on suicide attempt across age of onset among young men (natural cubic splines)*. The dotted line represents the ORs after controlling for offspring’s own inpatient care due to psychiatric diagnoses; *adjusted for parental socioeconomic status, civil status, suicidal behaviour, inpatient care due to psychiatric diagnoses, death due to other reasons than suicide and parental disability pension; “long”: Long-term was defined as an offspring’s suicide attempt occurring later than 2 years after exposure to parental markers of morbidity and mortality; “Parent psychiatric”: At least one parent in inpatient care due to a psychiatric diagnosis.

Table 2 and 3 present univariate and multivariate ORs for exposure to maternal and paternal markers of morbidity and mortality for female and male offspring, respectively. Figure 1 and 2 show those effects of exposure to parental makers which were statistically significant age dependent. Several additional significant age dependent effects indicated in Table 2 and 3 were difficult to interpret due to very wide confidence intervals and are therefore not presented in figures.

### Parental Suicide and Suicide Attempt

With regard to parental suicide, short term effects were associated with the highest odds ratios (OR 2.9) among girls, whereas exposure during childhood had the greatest impact on suicide attempt risk among boys (OR 3.0) in the crude analyses (Table 2 and 3). After adjustment for parental socioeconomic status and civil status, and all other parental exposures ORs decreased to 1.6 and 1.4, respectively. Futher adjusting for individual inpatient care due to psychiatric diagnoses (a potential mediator) the ORs were altered to 1.9 and 1.1, respectively, the latter not being significant any longer. With a mediated proportion of 82%, the effect of parental suicide during childhood among boys is therefore to a considerable degree mediated through individual inpatient care due to psychiatric diagnoses.

In the univariate analyses, short-term exposure to maternal suicide attempt was associated with the highest risk of suicide attempt among all exposures studied (OR 4.1 for both girls and boys) (Table 2 and 3). After multivariate adjustment odds of attempting suicide within 2 years after a maternal suicide attempt remained two-fold increased for both girls and boys. In fully adjusted analyses exposure during childhood and long term effects of exposure to maternal suicide attempt resulted in ORs of 1.6 to 1.7 for both sexes. Decreases in estimates were particularly due to adjustments for other parental risk factors. Similar patterns could be observed for exposure to paternal suicide attempt except that exposure to paternal suicide attempt during childhood was associated with higher ORs than long term exposure, particularly for boys (Table 3). Considering the effect of individual inpatient care due to psychiatric diagnoses (mediated proportion: 64%) in the analyses of exposure to paternal suicide attempt during childhood among boys decreased the estimate considerably. For girls, the effect of exposure to paternal suicide attempt during childhood decreased significantly with age, from a more than threefold increased risk at the age of 20 to an odds ratio of 1.4 at the age of 30 (Figure 1A, p = 0.042 for the null hypothesis that the effect is constant across ages). This significant variation with age was lost after controlling for offspring’s inpatient care due to psychiatric diagnoses.

### Parental Inpatient Care Due to Psychiatric Diagnoses

Short-term effects of exposure to maternal inpatient care due to psychiatric diagnoses were much more detrimental with regard to the risks of carrying out a suicide attempt among young women compared to young men (multivariate adjusted ORs reached 2.6 and 1.4, respectively; p = 0.001 for gender difference) (Table 2 and 3). Considerable lower short-term effects for girls in case of paternal compared to maternal inpatient care due to psychiatric diagnoses were found (Table 2). Effects during childhood were stronger than short- and long-term effects in case of exposure to paternal inpatient care due to psychiatric diagnoses for both young women and men (Table 2 and 3, Model II). Adjustments for parental socio-economic and civil status as well as parental suicidal behavior had considerable effects on the estimates.

Gender differences were also seen with respect to age modification of the short- and long-term effects of parental inpatient care due to psychiatric diagnoses on offspring risk of suicide attempt (Figure 1 C and 2 A). Short-term effects as opposed to long-tem effects of exposure to parental inpatient care decreased significantly with age among young women compared to young men, respectively (p = 0.0015 and p = 0.022 for the null hypothesis that the effect is constant across ages, Figure 1 C and Figure 2 A). The mediated proportions by offspring’s own inpatient care due to psychiatric diagnoses were 39% and 8%, respectively. This variation with age remained significant for both analyses after taking account of offspring’s own inpatient care due to psychiatric diagnoses. The effects of short-term exposure to parental inpatient care decreased strongly from an approximately four fold elevated risk around the age of 17 years to a two fold increase between 20 and 25 years of age, followed by a further decrease to practically no risk increase when the young women reached the age of 30 (Figure 1). Long-term effects of parental inpatient care due to psychiatric diagnoses decreased from an approximately three-fold increased risk around the age of 17 to a two-fold increase around 30 years of age in young men (Figure 2).

### Disability Pension

Both exposure to maternal and paternal disability pension due to psychiatric diagnoses showed strongest effect sizes for short-term exposure in the analyses adjusting for all other parental risk factors among both young women and men (ORs ranging from 1.3 to 1.5). None of these associations remained statistically significant after considering the mediating effect of offspring’s inpatient care due to psychiatric diagnoses, with exception of the long-term exposure to parental disability pension due to psychiatric diagnoses in young women. With regard to maternal and paternal disability pension due to somatic diagnoses, long-term effects and effects of exposure during childhood on offspring’s suicide attempt risk were stronger than short-term effects for girls and boys (data not shown). Short term effects of maternal disability pension due to somatic diagnoses decreased significantly with age at suicide attempt in young women, leading to no risk after the age of 27 (p = 0.006 for variation with age) (Figure 1 B). The mediated proportion of offspring’s inpatient care due to psychiatric diagnoses was 45%. The variation with age remained here significant after taking account of offspring’s own inpatient care due to psychiatric diagnoses.

### Death Other than Suicide

With regard to maternal and paternal death due to other causes than suicide, significantly increased ORs could be found only for young men for short-term effects of exposure to maternal death (OR 1.5) and long-term effects of exposure to paternal death (OR 1.2), adjusted for all parental risk factors. Particularly adjustment due to socio-economic and civil status decreased the effect on the estimates related to parental death due to other cases than suicide. Mediation through own psychiatric morbidity had only a marginal effect.

## Discussion

### Main Findings

Different patterns for short- and long-term effects and effects of exposure during childhood for suicide attempt in offspring were found with regard to various markers of maternal and paternal morbidity and mortality among young women and men. Short-term effects were generally associated with higher ORs than long term effects, particularly among girls. Short -term effects of exposure to maternal suicide attempt were among the strongest risk factors, associated with two-fold increased ORs for suicide attempt in young women and men after multivariate adjustment. Exposure to maternal markers of morbidity and mortality was mainly associated with higher risk estimates than exposure to paternal markers. Some gender differences were found particularly with regard to short-term effects of exposure to maternal inpatient care due to psychiatric diagnoses, being more detrimental for young women. With regard to exposure to parental inpatient care due to psychiatric diagnoses, short-term effects decreased significantly with age for young women. For young men, long-term effects of exposure to parental inpatient care due to psychiatric diagnoses decreased significantly with age.

### Methodology

One of the main strengths of this study include the use of register data with good validity [20], [27], [28]. For exemple the loss of individual information in the NPR is less than 1% since 1985 [20]. An additional strength is the longitudinal data over a period of more than 30 years assuring similar coverage of parental and offspring’s measures of psychopathology and suicide attempt. Furthermore, the large number of cases which allowed detailed analyses of short- and long-term exposure to both maternal and paternal markers of morbidity and mortality should be stressed. The present study was not affected by recall bias, a frequent problem in data derived from clinical settings. Limitations of the present study include that data on maternal and paternal psychiatric inpatient care and suicide attempt as well as offspring’ s suicide attempt did not cover all kinds of psychopathology as only psychiatric morbidity and suicide attempt resulting in hospitalization were registered. In particular, only the first diagnoses for parental psychiatric inpatient care were used for this study.

Studies from Europe and the US indicate that up to two thirds of individuals with diagnosable mental disorders do not receive treatment [29]. In Sweden, it was estimated that approximately 47% of suicide attempters in the general population do not seek medical treatment [30]. Therefore, both the exposure and the outcome measures derived from inpatient care data reflect most likely psychopathology and suicide attempts of considerable medical severity. Some residual confounding of unmeasured parental and offspring’s psychopathology and parental suicide attempts is therefore likely. The findings may be predominantly generalised to suicide attempts necessitating inpatient care in young individuals. We have assessed parental suicidal behaviour and psychopathology seperatly due to the evidence suggesting that familial suicidal behaviour is transmitted in families above and beyond the transmission of mental disorders [6]. A possible concern, however, could be that parental suicide is actually merely a subgroup among parents with mental disorder. Still, only 57% of parents with suicides also have had an inpatient care due to a psychiatric diagnosis. Thus, these exposures can be treated as different, albeit related, exposures.

### Short- term Effects of Exposure

Maternal markers of morbidity and mortality showed the strongest effect sizes if exposure was short-term among girls. Maternal suicide attempt showed the strongest effect if exposure was short-term for both girls and boys. This finding stresses the importance of maternal suicide attempt for offspring’s suicide attempt as a triggering factor as well as the possibility for an imitation effect. Studies to date have tried to disentangle the underlying mechanisms of familial transmission of suicidal behavior. Potential mechanisms include genetic predisposition, psychosocial environment and imitation [31]. Since previous studies have not found evidence of a temporal relationship between parental and offspring’s suicidal behavior consistent with imitation [31], our finding adds an interesting piece to this body of evidence. In future studies it seems to be important to scrutinize the effects of maternal and paternal suicidal behavior on suicidal behavior in female and male offspring separately in order to elucidate potential imitative effects. These detailed analyses require sufficient number of cases, one of the strengths of this study.

### Sex Differences

We found a significant sex difference related to the short-term exposure to maternal inpatient care due to psychiatric diagnoses on the risk of suicide attempt in offspring: the OR adjusted for other parental risk factors reached 2.6 and 1.4 in young women and men, respectively. Similar but not that considerable sex differences were reported earlier with regard to the risk of offspring’s suicide [15]. Our finding might suggest that exposure to maternal inpatient care due to psychiatric diagnoses as a risk factor for suicide attempt in offspring might be on a stronger emotional level for daughters than for sons. Exposure to maternal markers as compared to paternal was mainly associated with higher ORs for both girls and boys. These findings stress the importance of the mother as a role model and the strong attachment to the mother for both sexes and particularly for girls [32], [33].

### Exposure during Childhood

Some parental risk markers exerted the most detrimental effect if exposure occurred during early childhood as compared to short-term exposure to these risk markers, namely: for young women maternal and paternal disability pension due to somatic diagnoses and for young men paternal inpatient care due to psychiatric diagnoses. These analyses are expanding earlier reports of higher risk estimates of exposure to parental inpatient care due to psychiatric diagnoses and disability pension due to somatic diagnoses if exposure occurred in early childhood as opposed to later in life [12]. Further studies scrutinizing a potential stronger genetic vulnerability of particularly paternal as compared to maternal psychopathology for offspring suicide attempt and potential gender differences are warranted.

### Effect Modification by Age

This is to our best knowledge the first study analyzing if the effect of exposure to parental markers of morbidity and mortality, namely short- and long-term effects as well as effects of exposure during childhood, are modified by age. We found that short-term effects of exposure to parental inpatient care due to psychiatric diagnoses and maternal disability pension due to somatic diagnoses decreased significantly with age for young women, being associated with the highest risk in adolescence. Adolescence is associated with a higher degree of impulsive behavior and emotional turmoil than adulthood [14]. Teen suicidal behavior is also known to be characterized by a brief stress suicide interval and a lower level of planning and suicidal intent than adult suicidal behavior [34]. It is therefore of clinical importance to consider that the short-term risk for suicide attempt for girls in case of parental inpatient care due to psychiatric diagnoses is particularly high during adolescence.

The effect of exposure to a further parental marker decreased with age at offspring’s suicide attempt, namely the long-term effects of parental inpatient care due to psychiatric diagnoses for young men. These associations were independent from the offspring’s own psychopathology, measured by previous inpatient care due to psychiatric diagnoses. These risk factors might here resemble a stronger, maybe genetic, vulnerability for suicide attempt and their occurrence might be associated with an earlier age at suicidal behavior. Different familial risk factors, like a stronger familial loading for suicidal behavior and higher levels of impulsive aggression, have been reported to be associated with an earlier age at suicidal behavior [8], [9]. While additional studies are warranted to further scrutinize the particular importance of early and long-term exposure to parental psychopathology, clinicians should be aware of the particularly high risk for suicide attempt associated with these risk factors during adolescence.

### Conclusion

In general, parental morbidity and mortality were associated with an increase in suicide attempt risk in the offspring. The strongest risk for attempting suicide was within the two-year period following a mother’s inpatient care due to suicide attempt for both daughters and sons and mother’s inpatient care due to psychiatric diagnoses particularly for daughters. Adolescence represented the most critical period for attempting suicide when exposed to parental psychopathology. Preventive clinical and public health measures are strongly needed for adolescents shortly after they have been exposed to their parents’ inpatient care due to psychiatric diagnoses and suicidal behaviour. Effective prevention requires a tight intersectioral collaboration particularly collaboration between adult- and child-psychiatry. Examples of effective intervention for children of parents suffering from mental disorders have been published [35] and could be applied more broadly.
